# A real-world safety evaluation of new oral anticoagulants in elderly patients: evidence from the Adverse Drug Reaction Monitoring Center of Henan Province

**DOI:** 10.1080/20523211.2025.2547678

**Published:** 2025-09-02

**Authors:** Dexian Ma, Chen Chen, Mingyang Sun, Jie Chen, Weigao Cheng, Jiajing Cao, Ming Xia, Youhong Hu, Zhiyong Sun, Xuedong Jia, Zhao Yin

**Affiliations:** a Center for Drug Reevaluation of Henan, Zhengzhou, People’s Republic of China; b Department of pharmacy, The First Affiliated Hospital of Zhengzhou University, Zhengzhou, People’s Republic of China; c Department of Information Systems, Hanyang University Industrial Center, Seoul, Korea; d School of Chemistry and Chemical Engineering, Henan Normal University, Xinxiang, People’s Republic of China

**Keywords:** Novel oral anticoagulants, rivaroxaban, dabigatran etexilate, elderly patients, adverse reactions

## Abstract

**Background:**

The use of new oral anticoagulants (NOACs) is becoming increasingly widespread, but data on their adverse reactions are still incomplete. Further analysis based on data from the Drug Adverse Reaction Center is needed to guide safe clinical use.

**Methods:**

A retrospective analysis was performed on 281 cases of rivaroxaban and 48 cases of dabigatran etexilate-related ADR reported by medical institutions collected by a provincial Food and Drug Administration from 2018 to 2023.

**Results:**

Of the 329 ADRs, 164 males and 165 females were reported. Among the rivaroxaban-related ADRs, 271 were administered orally, 6 were given nasogastric feeding, 2 were given tube feeding, and 2 were intravenously instilled. Among the ADRs associated with dabigatran etexilate, 48 cases were administered orally. Serious adverse drug reactions were reported in 21.6% of cases (71 out of 330). The clinical manifestations of ADR of NOACs mainly include blood in the stool, blood in the urine, bleeding gums, coagulation disorders and ecchymosis. The causal relationship between serious adverse reactions and drugs was judged to be very likely in 24, 43 cases was judged to be probable, and 4 cases were unknown.

**Conclusions:**

Attention should be paid to the clinical use of NOACs in elderly patients, and pharmacovigilance should be strengthened, and the implementation of individualised medication regimen should be used to promote clinical safety and rational drug use.

## 1. Background

Thromboembolic disorders, including atrial fibrillation (AF), deep vein thrombosis (DVT), and pulmonary embolism (PE), are highly prevalent among elderly populations and are associated with substantial morbidity and mortality (Kimonge, 2023). Oral anticoagulants (OACs) are the cornerstone of prevention and treatment for these conditions, significantly reducing the risks of stroke, systemic embolism, and venous thromboembolism recurrence (Galanti et al., 2024). Traditionally, vitamin K antagonists (VKAs) such as warfarin have been widely used. Compared to VKAs, NOACs show a significant advantage in reducing the risk of intracranial hemorrhage (ICH). Studies have shown that dabigatran, apixaban, and edoxaban can reduce the risk of ICH by more than 50%, while rivaroxaban reduces it by approximately 20% (Decaix et al., 2024). In addition, NOACs have more predictable pharmacokinetic profiles, fewer drug and food interactions, and do not require routine coagulation monitoring (Lucà et al., 2023). They also appear to have a slight advantage in reducing all-cause mortality.

Nonetheless, anticoagulant therapy in elderly patients remains challenging due to age-related physiological changes, including reduced hepatic and renal function and decreased drug metabolism capacity, which can heighten the risk of adverse drug reactions (ADRs) (Valeriani et al., 2024). Warfarin’s narrow therapeutic index and its interactions with diet and concomitant medications often lead to fluctuations in the international normalised ratio (INR), increasing the risk of both bleeding and thrombosis (Gao et al., 2024; Morris et al., 2023). There are significant differences in the adverse event profiles of NOACs compared to warfarin in elderly patients (Pedro et al., 2024; Sakamoto et al., 2024). Most notably, the proportion of serious bleeding events caused by NOACs is significantly lower. Furthermore, results from network meta-analyses indicate that rivaroxaban is associated with a higher risk of clinically relevant non-major bleeding (CRNMB) and minor bleeding than dabigatran when used for the prevention and treatment of venous thromboembolism (VTE). Dabigatran, in most analyses, shows a moderate level of bleeding risk. Both agents carry a higher risk of gastrointestinal bleeding compared to apixaban, which warrants particular attention in older patients (Shahbar et al., 2025). Although NOACs are more convenient, renal impairment common in elderly patients can lead to drug accumulation and a higher risk of bleeding. Balancing efficacy and safety of anticoagulation therapy in elderly patients, therefore, remains a critical concern in clinical practice (Stuby et al., 2024).

While randomised controlled trials (RCTs) are the gold standard for evaluating drug efficacy and safety, their strict inclusion and exclusion criteria often exclude frail elderly patients with multiple comorbidities and polypharmacy, limiting the generalis ability of their findings (Kholmukhamedov et al., 2025). In contrast, real-world data can provide valuable insights into the safety and effectiveness of anticoagulants in routine clinical practice, especially among elderly patients with complex clinical profiles (Roberti et al., 2021). Analysis of spontaneous ADR reports from provincial pharmacovigilance databases can identify ADR patterns, high-risk populations, and potential contributing factors, thereby supporting evidence-based clinical decision-making. This study aims to evaluate the safety profiles of different anticoagulants in elderly patients using spontaneous ADR reports from the Henan Province Adverse Drug Reaction Monitoring Center. Furthermore, it seeks to identify potential risk factors associated with serious ADRs, providing evidence to inform individualised anticoagulant prescribing and guide regulatory pharmacovigilance strategies.

## 2. Methods

### 2.1. Data collection

Data were obtained from the spontaneous adverse drug reaction (ADR) reporting system of the Henan Province Adverse Drug Reaction Monitoring Center. The dataset includes basic report information, patient demographic data (age, sex, smoking and drinking history, allergy history), patient medical history, medication information, ADR details (System Organ Class [SOC] classification and clinical manifestations, severity, outcomes, and causality assessment), and report quality attributes. Data were cleaned and pre-processed according to the following inclusion and exclusion criteria. Inclusion criteria: (1) ADR reports related to NOACs between 2018 and 2023; (2) Patients aged ≥60 years; (3) Reports in which the causality between the ADR and NOACs use was assessed as ‘certain,’ ‘probable,’ or ‘possible.’ (Agbabiaka et al., 2008). Exclusion criteria: (1) Reports lacking critical information, such as age, drug name, or specific ADR details; (2) Reports not involving NOACs or those in which NOACs were only recorded as concomitant medications; (3) Reports in which the causality assessment between OAC use and ADR was categorised as ‘unlikely’ or ‘impossible.’ Due to the relatively small number of cases included in this study, the research team conducted manual screening to identify and eliminate duplicate entries.

### 2.2. Data setting

In this study, NOACs refer to rivaroxaban and dabigatran etexilate. Due to the lack of standardised ADR nomenclature within the reporting system, this study categorised ADRs according to the System Organ Class (SOC) classification from the Medical Dictionary for Regulatory Activities (MedDRA). Based on the Chinese ‘Regulations on Adverse Drug Reaction Reporting and Monitoring’, ADRs were classified as either serious or non-serious (Bai et al., 2022). An ADR was defined as serious if it resulted in any of the following outcomes: death; teratogenicity, carcinogenicity, or congenital defects; permanent disability; permanent organ dysfunction; hospitalisation or prolongation of hospitalisation; or any other clinically significant condition. Cases that did not meet these criteria were classified as non-serious ADRs. The causality of ADRs was assessed using the WHO-UMC criteria, which classifies the likelihood of an ADR as certain, probable, possible, unlikely, conditional/unclassified, or unassessable (Manjhi et al., 2024).

### 2.3. Statistical analysis

Statistical analyses were conducted using IBM SPSS version 24.0 (IBM, United States) and Microsoft Excel. Descriptive analyses were performed for variables including year of report, patient age, sex, proportion of serious ADRs, and ADR outcomes. Categorical variables were presented as frequencies and percentages. The chi-square test or Fisher’s exact test was used for comparisons between groups. For comparisons involving more than two groups – such as stratified age groups, different medication dosages, and different seasons of medication use – the Kruskal–Wallis H test was applied. Logistic regression analysis was performed to identify factors associated with the occurrence of serious ADRs. A *p*-value <0.05 was considered statistically significant. When performing multiple comparisons, the significance level will be adjusted accordingly.

## 3. Results

### 3.1. Sample characteristics

A total of 329 ADR reports related to NOACs were collected, including 281 associated with rivaroxaban and 48 with dabigatran etexilate. Among these, 78.4% (258/329) of participants experienced ADRs of mild to moderate severity, while 21.6% (71/329) were classified as serious. No fatal cases were reported. Participant ages ranged from 60 to 97 years, with a mean of 77.25 ± 3.6 years. Participants were stratified into three age groups (60–69, 70–79, and ≥80 years), and a statistically significant difference in the distribution of SADRs was observed across age groups (*p* < 0.05), with a higher incidence noted among those aged ≥80 years (*p* < 0.0125). Of the total sample, 164 were male and 165 were female. No significant difference in ADR incidence between sexes was observed. In addition, 28.27% of participants suffered from more than one disease before the ADR occurred. A small proportion had a family history of ADRs, engaged in smoking or alcohol consumption, or were exposed to polypharmacy. Detailed demographic characteristics are presented in Table 1.

**Table 1.** Number and proportion of serious and non-serious reports by personal characteristics.

Characteristics	Serious N (%)	Non-serious N (%)	Total	*p*-Value
*Age* (refer to 60-69)				
60–69	13 (15.1%)	73 (84.9%)	86	
70–79	23 (17.4%)	109 (82.6%)	132	0.010
≥80	35 (31.5%)	76 (68.5%)	111	0.007*
*Gender*				0.675
Male	36 (22%)	128 (78%)	164	
Female	34 (20.6%)	131 (79.4%)	165	
*Original disease*				0.011*
1	50(21.2%)	186 (78.8%)	236	
≥2	21(22.6%)	72(77.4%)	93	
*ADR history*				0.642
Yes	0 (0%)	6 (100%)	6	
No	71 (22%)	252 (78%)	323	
*Drinking or Smoking*				0.218
Yes	7 (21.9%)	25 (78.1%)	32	
No	64 (21.5%)	234(78.5%)	298	
*Polypharmacy*				0.759
Yes	3(25.0%)	9(75.0%)	12	
No	75(23.7%)	242(25.0%)	317	

### 3.2. Yearly distribution and trend of ADR severity

**Figure 1.**
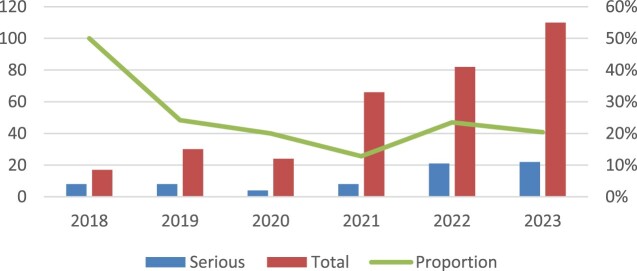
Number of reports and the proportion of SADR reports in each year.

Figure 1 illustrates the temporal trend of ADRs reported from 2018 to 2023. The annual number of both SADR and non-SADR cases showed an overall increasing pattern. In 2018, the proportion of SADRs was relatively high; however, in subsequent years, the proportion remained generally stable. No statistically significant difference was observed in the yearly distribution of SADRs (*p* = 0.218).

### 3.3. System organ classification and clinical manifestations of ADRs

ADRs were coded using MedDRA version 27.1. The system organ classes (SOCs) and corresponding clinical manifestations associated with anticoagulant-induced ADRs are summarised in Table 2. Notably, several ADRs not previously listed in the drug labelling were identified, including tremor, chest tightness, pain, polyuria, abdominal distension, flushing, scleral hemorrhage, eye hemorrhage, retinal hemorrhage, prolonged activated partial thromboplastin time, granulocytopenia, leukopenia, and abnormal breath sounds, which warrant further attention. Among the 382 reported ADR manifestations, bleeding was the most common clinical presentation (e.g. hematochezia in 47 cases, gingival bleeding in 42 cases, and hematuria in 35 cases). Of these, hematochezia accounted for the largest number of SADRs (20 cases). Gastrointestinal symptoms were the second most frequently reported (e.g. gastrointestinal hemorrhage in 18 cases, nausea in 10, abdominal pain in 6, vomiting in 6), followed by allergic reactions (e.g. rash in 13 cases). Details are provided in Table 2.

**Table 2.** Systemic organ classification and clinical manifestations of ADR caused by oral anticoagulants.

SOC	Case Count (%)	ADR (Case Description)	Severe Cases (%)	Clinical Manifestations
Various Examinations	25 (6.5%)	Prolonged coagulation time (13), Abnormal prothrombin time (2), Prolonged aPTT (1), Thrombocytopenia (5), Granulocytopenia (1), Leukopenia (1), Abnormal CBC (1), Abnormal breath sounds (1)	6 (6.7%)	Prolonged coagulation time <4 cases, Granulocytopenia (1), Leukopenia (1)
Neurological Disorders	5 (1.3%)	Cerebral hemorrhage (1), Dizziness (3), Tremor (1)		Prolonged coagulation time (4), Granulocytopenia (1), Leukopenia (1)
Respiratory, Thoracic and Mediastinal Disorders	26 (6.8%)	Epistaxis (17), Pulmonary hemorrhage (1), Hemoptysis (8)		
Skin and Subcutaneous Tissue Disorders	44 (11.5%)	Ecchymosis (22), Rash (13), Red papules (4), Blisters (1), Pruritus (4)	2 (2.2%)	Dizziness (1), Cerebral hemorrhage (1)
General Disorders and Administration Site Conditions	5 (1.3%)	Arm swelling (1), Chest tightness (2), Chest swelling/mass (1), Pain (1)	1 (1.1%)	Red papules (1)
Renal and Urinary Disorders	39 (10.2%)	Hematuria (35), Bladder hemorrhage (1), Urinary tract bleeding (1), Polyuria (1), Worsened renal function (1)	1 (1.1%)	Chest tightness (1)
Gastrointestinal Disorders	180 (47.1%)	Oral bleeding (10), Nausea (10), Abdominal pain (6), Vomiting (6), Acid reflux (4), Diarrhea (3), Indigestion (1), Abdominal distension (1), Abdominal discomfort (3), Hematochezia (47), Upper GI bleeding (10), Lower GI bleeding (8), Hematemesis (3), GI bleeding (15), Hemoptysis (11)	10 (11.1%)	Hematuria (9), Urinary tract bleeding (1)
Vascular and Lymphatic Disorders	45 (11.8%)	Gingival bleeding (42), Venous thrombosis (1), Subcutaneous bleeding (19), Hematoma (1), Flushing (1), Coagulation disorders (21), Anemia (2)	46 (51.1%)	Hematochezia (20), Nausea (1), Upper GI bleeding (7), Hematemesis (1), Lower GI bleeding (3), Diarrhea (1), GI bleeding (8), Hemoptysis (3)
Eye Disorders	3 (0.8%)	Scleral hemorrhage (1), Eye bleeding (1), Retinal hemorrhage (1)	17 (18.9%)	Coagulation disorder (8), Gingival bleeding (2), Subcutaneous bleeding (6), Flushing (1), Anemia (2)
Hepatobiliary Disorders	2 (0.5%)	Abnormal liver function (2)	2 (2.2%)	Scleral hemorrhage, Eye bleeding, Retinal hemorrhage (1), Eye congestion (1)
Psychiatric Disorders	5 (1.3%)	Fatigue (5)	1 (1.1%)	Abnormal liver function (1)
Cardiac Disorders	2 (0.5%)	Palpitations (2)	3 (3.3%)	Fatigue (3)
Injuries, Poisoning and Procedural Complications	1 (0.3%)	Abdominal tenderness (1)	1 (1.1%)	Palpitations (1)

### 3.4. Frequently reported ADRs

Table 3 presents the top 10 types of ADR symptoms observed among the participants. A total of 329 participants developed a variety of ADRs, with the most frequent symptoms involving various forms of bleeding. Gingival bleeding was the most common (42 cases), followed by hematuria (35), ecchymosis (22), coagulation disorders (21), subcutaneous hemorrhage (19), epistaxis (17), gastrointestinal bleeding (15), prolonged coagulation time (13), rash (13), and hemoptysis (11). These ADRs primarily affected the hematologic, gastrointestinal, and integumentary systems and may have important clinical implications, particularly in relation to bleeding risk.

**Table 3.** Number and proportion of ADRs and SADRs (Top 10).

Rank	ADR Symptom (n = 208)	Number of Cases	Proportion (%)
1	Gingival bleeding	42	20.19
2	Hematuria	35	16.83
3	Ecchymosis	22	10.58
4	Coagulation disorder	21	10.10
5	Subcutaneous hemorrhage	19	9.13
6	Epistaxis	17	8.17
7	Gastrointestinal bleeding	15	7.21
8	Prolonged coagulation time	13	6.25
9	Rash	13	6.25
10	Hemoptysis	11	5.29

### 3.5. Medication characteristics

Table 4 presents the association between medication-related factors and the severity of ADRs. Regarding seasonal variation, participants who received medications in summer had a significantly lower incidence of SADRs compared to those in winter (*p* = 0.007), while no statistically significant differences were observed in spring or autumn. In terms of medication dosage, participants who received rivaroxaban 10 mg/day showed a significantly lower proportion of SADRs compared to those receiving 20 mg/day (*p* = 0.006). No significant differences in SADR occurrence were observed for rivaroxaban 15 mg/day (*p* = 0.109) or for different doses of dabigatran etexilate (*p* = 0.495). Additionally, hospital level and region of origin had no significant impact on the incidence of SADRs.

**Table 4.** Number and proportion of serious and non-serious reports by medication characteristics.

Characteristics	Serious N (%)	Non-serious N (%)	Total	*p*-Value
*Medication season (Refers to Winter)*				
Spring	18	64	82	0.214
Summer	10	68	78	0.007*
Autumn	22	79	101	0.183
Winter	21	47	68	
*Medication dosage*				
Rivaroxaban (Refers to 10 mg/d)				
10 mg/d	4	39	43	
15 mg/d	14	53	67	0.109
20 mg/d	38	88	126	0.006*
Dabigatran Etexilate				0.495
220 mg/d	10	23	33	
300 mg/d	1	5	6	

### 3.6. Causality assessment categories

Table 5 shows the distribution of serious and non-serious ADR reports based on causality assessment categories. A statistically significant difference was observed between the severity of ADRs and the level of causality assessment (*p* < 0.0001). Specifically, a higher proportion of serious ADRs was classified as ‘Possible’ (43/105), whereas the majority of non-serious ADRs were categorised as ‘Probable’ (175/199). No significant difference was found in the ‘Certain’ category (*p* = 0.523), likely due to the small number of cases (n = 4).

**Table 5.** Number and proportion of serious and non-serious reports by causality assessment categories.

Causality assessment	Serious N (%)	Non-serious N (%)	Total	*p*-Value
Possible(refers to possible)	43	62	105	
Probable	24	175	199	＜0.0001*
Certain	1	3	4	0.523

### 3.7. Outcomes of ADRs

Among all reported cases, complete recovery was observed in 83 participants, improvement in 223 cases, no improvement in 6 cases, and outcomes were unspecified in 17 cases. In the subgroup of serious ADRs, 18 cases resulted in full recovery, 48 showed clinical improvement, 1 case exhibited no improvement, and 4 cases lacked sufficient follow-up information to determine the outcome.

## 4. Discussion

To the best of our knowledge, this study is the first in China to investigate the safety profile of NOACs in elderly patients based on data from the Henan Provincial Adverse Drug Reaction Monitoring Center. The mean age of the study population was 77 years, with 95.4% (314/329) of participants aged between 69 and 89 years, and 4.6% (15/329) aged 90 years or older. These findings are consistent with previous research (Wu et al., 2024), which reported that the most common age group was above 65 years. Moreover, the age distribution in our study aligns with existing epidemiological data, indicating that individuals aged 60 years and above account for more than 60% of patients with thromboembolic diseases, and this proportion exceeds 70% in specific subtypes such as pulmonary embolism and ischemic stroke (Lutsey & Zakai, 2023).

The findings of this study indicate a statistically significant association between age and the incidence of serious adverse drug reactions (SADRs) among elderly patients receiving NOACs. Participants aged ≥80 years exhibited a significantly higher incidence of SADRs compared to those aged 60–69 and 70–79 years (*p* < 0.0125). This result is consistent with previous pharmacovigilance and clinical studies, suggesting that advanced age may serve as an independent risk factor for increased susceptibility to adverse reactions associated with anticoagulant therapy (Zhao et al., 2025). Meanwhile, it was shown that the risk of bleeding in elderly individuals is approximately 1.53 times higher than in younger populations due to age-related deterioration of organ function (Udomnilobol et al., 2024). Besides, comorbid conditions such as hypertension and diabetes are common among the elderly, and polypharmacy is frequently encountered. Our study observed a trend toward increased SADR incidence in patients receiving multiple medications, although the difference was not statistically significant. This may be due to data incompleteness or limited sample size. Nonetheless, previous studies have identified polypharmacy as a significant risk factor for SADRs (Jiao et al., 2021; Yan et al., 2022).

The elevated risk of SADRs in the elderly may be attributed to a combination of physiological and pharmacological factors, including age-related decline in organ function, reduced metabolic capacity, and frequent polypharmacy (Rodrigues et al., 2021). Rivaroxaban has an oral bioavailability of approximately 80–100%, with dose-dependent saturable absorption. In elderly patients, impaired gastrointestinal function may compromise drug absorption and increase the risk of ADRs. In the present study, a higher proportion of SADRs was observed in participants receiving the 20 mg dose of rivaroxaban, indicating that dosage may be a critical factor influencing ADR severity (Zazzara et al., 2021). Physiological aging alters drug metabolism and increases ADR risk. Reduced renal clearance, especially when creatinine clearance is <50 mL/min, prolongs drug exposure and raises bleeding risk. Hepatic changes, including decreased blood flow and CYP3A4 activity, may impair rivaroxaban metabolism (Tesfaye et al., 2024). Vascular fragility and impaired coagulation, both common in the elderly, further predispose patients to bleeding events such as intracranial or subcutaneous hemorrhage (Huang et al., 2024). Moreover, drug-drug interactions related to metabolic pathways significantly influence the incidence and severity of ADRs in elderly patients receiving NOACs. Both rivaroxaban and apixaban are substrates of CYP3A4 and P-glycoprotein (P-gp); co-administration with potent inhibitors such as ketoconazole, erythromycin, or verapamil can elevate plasma drug concentrations, thereby increasing the risk of bleeding events. In contrast, edoxaban is primarily affected by P-gp and undergoes relatively less hepatic metabolism (Ferri et al., 2022). When using NOACs in older adults, particularly the very elderly, close monitoring of liver and kidney function is essential (Zhang et al., 2021).

This study found that bleeding was the most common adverse reaction among elderly patients receiving NOACs, primarily manifesting as gastrointestinal bleeding (e.g. melena, hematochezia), gingival bleeding, hematuria, and bruising. Hematochezia accounted for 20.2% (20/90) of all serious ADRs, underscoring its clinical importance (Candeloro et al., 2023). Elderly patients often experience gastrointestinal symptoms while taking dabigatran or rivaroxaban, including bleeding, nausea, vomiting, and abdominal pain. In this study, serious gastrointestinal ADRs included eight cases of GI bleeding, three of hemoptysis, and one case each of nausea, hematemesis, and diarrhea. These events may be attributed to age-related changes and drug properties. In older adults, gastric mucosa is thinner and less perfused, increasing sensitivity to irritation (Benamouzig et al., 2022). Slower intestinal motility prolongs mucosal drug contact, while reduced renal function leads to drug accumulation, especially for renally excreted agents like dabigatran (Zhang et al., 2021). Dabigatran contains tartaric acid, which can irritate the stomach lining. Rivaroxaban inhibits factor Xa, and dabigatran blocks thrombin, both impairing coagulation and mucosal healing. Dabigatran’s partial biliary excretion may disrupt gut flora or mucosal barriers, potentially causing diarrhea (Kocis et al., 2024). Co-administration with NSAIDs (e.g. ibuprofen) or antiplatelets (e.g. aspirin) further increases bleeding risk. In bleeding cases, NOACs should be discontinued. Given their short half-life, coagulation function typically recovers within 12–24 hours (Seshan, 2024). Although allergic reactions are relatively rare, their potential risk may be significantly elevated due to multiple complex factors. In this study, serious allergic reactions included one case of erythematous papules. Such events likely result from the interplay of age-related physiological vulnerability, altered drug metabolism, and immunosenescence in the elderly population (Wu & Ma, 2021).

This study identified a significant association between ADR severity and causality assessment levels. Serious ADRs were more often classified as ‘Possible,’ while non-serious ADRs were more frequently assessed as ‘Probable’ (*p* < 0.0001). This suggests that clinical severity may influence the confidence of causality judgments (Srisuriyachanchai et al., 2023). Serious ADRs often occur in complex clinical contexts, such as polypharmacy and comorbidities, which increase diagnostic uncertainty (McGettigan et al., 2024). In such cases, reporters may adopt a conservative stance, opting for ‘Possible’ to avoid over-attribution in the absence of definitive evidence (Xia et al., 2024). In contrast, non-serious ADRs are typically easier to assess due to clearer temporal relationships and fewer confounding factors. The ‘Certain’ category was rarely used (only 4 cases), with no significant difference across severity groups (*p* = 0.523). This likely reflects the high threshold required for ‘Certain,’ which demands strong evidence, including dechallenge and rechallenge data, and exclusion of alternative causes (Lucas et al., 2022). These findings align with our prior research showing that clinicians often underreport or downgrade causality due to concerns about complexity, workload, or accountability (Xia et al., 2024). The tendency toward conservative reporting may lead to underestimation of drug-related risks. Incorporating structured tools like the Naranjo algorithm may help improve the consistency of ADR assessment and enhance signal detection reliability (Bereda, 2021). Notably, the results of the present study showed that SADRs were less frequent in summer, which may be indirectly attributed to seasonal factors such as temperature, which could influence medication adherence of elderly patients (Chodick et al., 2024).

This study utilised real-world data from the Henan Provincial Adverse Drug Reaction (ADR) Monitoring System to characterise the clinical profile of NOAC-related ADRs among elderly patients, a population particularly vulnerable to adverse outcomes. The results offer meaningful insights into the safety of anticoagulant use in routine clinical settings and provide a practical reference for improving risk management in geriatric anticoagulation therapy. In addition to evaluating the types and severity of ADRs, the study further explored relevant factors including dosage, route of administration, and age group distributions, enabling a more comprehensive risk assessment. However, several limitations should be acknowledged. First, the data were derived from a single provincial monitoring centre, which may restrict the generalizability of the findings to broader national contexts. Second, reliance on a spontaneous reporting system is inherently subject to reporting bias, and variable data quality, potentially leading to an underestimation of the true incidence of ADRs and serious ADRs (SADRs). Third, the database lacked key clinical variables, such as detailed records of concomitant medications, laboratory findings, and precise timelines of drug administration, which limited the depth of mechanistic and stratified risk analyses. Given the missing data, the potential impact of confounding factors such as drug–drug interactions and renal impairment could not be evaluated. Moreover, the absence of denominator data prevented the estimation of incidence rates and hindered comparative risk evaluation across different medications or patient subgroups.

## 5. Conclusion

Based on current evidence, NOACs represent a preferable anticoagulation option for elderly patients, particularly those of advanced age or with impaired renal function. However, the associated bleeding risk–especially gastrointestinal bleeding–remains a major clinical concern. Given that elderly individuals often present with polypharmacy, declining organ function, and complex comorbidities, a comprehensive pre-treatment evaluation of age, body weight, renal function, and concomitant medications is essential to guide appropriate regimen selection and avoid unsubstantiated dose adjustments. To mitigate the risk of adverse events, regular monitoring of renal function and potential drug–drug interactions is recommended, along with enhanced adherence management and, when necessary, therapeutic drug monitoring. Furthermore, ongoing strengthening of pharmacovigilance systems and adverse drug reaction reporting mechanisms is critical to ensuring the safe and effective use of NOACs in aging populations.

## Data Availability

Data are available on reasonable request. The thematic data that support the findings of this present study are available from the corresponding author on reasonable request.
